# The Odor of Iodoform

**Published:** 1882-11

**Authors:** 


### The Odor of Iodoform.

Having tried nearly all the devices that have been suggested for mitigating or disguising the odor of iodoform, and found them all of little or no avail, we have lately come nearer to the object by using oil of eucalyptus, according to the following formula:

℞ Pulv. iodoform., ℥ ss.;

Ol. eucalypti, f ʒ ss.;

Vaselin., ℥ iv.

M., fiat unguentum.

We do not remember to have seen any account of the oil having been used for this purpose by others. The ointment thus prepared is not without odor, but the odor is not that of iodoform. —*N. Y. Med. Jour. and Obst. Rev.*

